# Targeting urinary tract infections: Anti-MDR efficacy of Syzygium aromaticum small molecules against uropathogenic Escherichia coli through in vitro and in silico approaches

**DOI:** 10.3205/000363

**Published:** 2026-07-01

**Authors:** Farhan Kesawa Mukti, Fairuz Fajriani Kusumaningrum, Muhammad Evy Prastiyanto, Daniel Geleta, Abdul Rahman Siregar, Wahyu Aristyaning Putri

**Affiliations:** 1 Department of Tropical Biology, Faculty of Biology, Universitas Gadjah Mada, Yogyakarta, Indonesia; 2 Department of Medical Laboratory Technology, Faculty of Health and Nursing, Universitas Muhammadiyah Semarang, Semarang, Indonesia; 3 Department of Medical Laboratory Sciences, Jimma University, Jimma, Oromia, Ethiopia

**Keywords:** clove, uropathogenic Escherichia coli, urinary tract infection, multidrug resistance, PBP2, AcrB

## Abstract

Urinary tract infections (UTIs) are becoming increasingly challenging to treat owing to the emergence of multidrug-resistant (MDR) strains, including uropathogenic *Escherichia coli* (UPEC). Clove (*Syzygium* *aromaticum* L.) has antimicrobial activity that can alter UPEC morphology and exhibit inhibitory effects. However, the mechanisms underlying the inhibition of specific small molecules remain largely unknown. This study aimed to explore the potential of small molecules in clove flower extract as anti-MDR UPEC agents using in vitro and in silico approaches. Extraction was performed using ethanol and n-hexane, yielding 27.44% and 9.9%, respectively. The ethanol extract exhibited inhibitory activity against MDR-UPEC, forming clear zones ranging from 11.07±0.32 (250 mg/mL) to 16.77±1.36 (1,000 mg/mL) in a disk diffusion assay. However, the combination of ethanol extract and ciprofloxacin resulted in antagonistic effects. GC-MS analysis of the ethanol extract identified 29 small molecules, which were subsequently analyzed using PASS Online, Lipinski’s Rule of Five, and molecular docking against target proteins PBP2 and AcrB. Three small molecules showed the potential to inhibit PBP2, and ten small molecules targeted AcrB. Spesifically, 8,14-Seco-3,19-epoxyandrostane-8,14-dione, 17-acetoxy-3β-methoxy-4,4-dimethyl-, Ethyl iso-allocholate and Estra-1,3,5(10)-trien-17β-ol show binding affinities of –7.9; –7.6; dan –8.0 kcal/mol to PBP2, and –9.3; –8.5; dan –8.6 kcal/mol to AcrB, respectively. These three small molecules are promising candidates for developing plant-based antibacterial agents against MDR UPEC.

## 1 Introduction

Urinary tract infections (UTIs) are among the most prevalent infections, with significant mortality, morbidity, and recurrence rates [1]. It is reported to affect women, with more than 50–60% experiencing it at least once in their lifetime [2]. In Indonesia, an estimated 180,000 new UTI are reported annually, highlighting the clinical burden of this condition [3]. Uropathogenic *Escherichia coli* (UPEC) is the leading causative agent of both uncomplicated and complicated UTIs [4], responsible for over 80% of cystitis and 70% of uncomplicated acute pyelonephritis in community-acquired cases [5].

The extensive use of antibiotics and lack of clinical investigations have contributed to the emergence of resistant strains [1]. Consequently, the management of UTI cases is becoming increasingly difficult owing to the rapid spread of antibiotic resistance among Gram-negative bacteria [6]. The emergence of multidrug-resistant (MDR) strains means that the choice of antibiotics for treating UTIs is limited [7]. MDR is defined as non-susceptibility to at least one agent in three or more antimicrobial categories [8]. Two resistance mechanisms were highlighted in this study: penicillin-binding proteins (PBPs) and the overexpression of efflux pump systems, such as AcrAB-TolC. PBPs catalyze peptidoglycan cross-linking during cell wall biosynthesis, and PBP2 is responsible for maintaining the bacterial rod shape during elongation [9], [10]. AcrB, a key component of the AcrAB-TolC efflux system, contributes significantly to MDR by activating TolC to export various antibiotics from bacterial cells [11].

Given the limitations of conventional antibiotics against MDR-UPEC [7], natural products have gained attention as alternative antimicrobial agents [12]. Medicinal plants, particularly those containing diverse secondary metabolites, are rich in bioactive molecules [13]. Clove (*Syzygium aromaticum* L.), a plant native to Indonesia, has long been used in traditional medicine for its antimicrobial, antifungal, and anti-inflammatory properties [1], [14]. Its essential oil is rich in eugenol and other phenolic compounds with documented antibacterial activity [1], [15].

Previous studies have shown that clove extract inhibits UPEC growth and disrupts bacterial cell morphology [1]. However, the specific mechanisms of action and active small molecules involved, particularly those targeting resistance-associated proteins, remain poorly understood. Therefore, this study aimed to evaluate the antibacterial activity of clove extract against MDR-UPEC, identify its small-molecule constituents using gas chromatography-mass spectrometry (GC-MS), and investigate their interactions with key resistance-related proteins (PBP2 and AcrB) through molecular docking. These findings are expected to provide new insights into the potential use of clove-derived small molecules as alternative therapeutics against multidrug-resistant uropathogenic *Escherichia* *coli*. 

## 2 Materials and methods

### 2.1 Bacterial strains and plant material

Clinical isolates of multidrug-resistant uropathogenic *Escherichia coli* (MDR-UPEC) were obtained from patients with UTI and provided by the Microbiology Laboratory, Faculty of Biology, Universitas Gadjah Mada, Yogyakarta, Indonesia. *E. coli* ATCC was used as a control. Fresh clove buds (*Syzygium aromaticum* L.) were collected from local farmers in Magetan, East Java, Indonesia. The plant material was air-dried before extraction.

### 2.2 Preparation of extract

Dried clove buds were ground into a powder and subjected to maceration using absolute ethanol (polar) or n-hexane (non-polar) as solvents in a 1:3 ratio (w/v) for 3×24 h at room temperature with occasional agitation. The filtrate was concentrated using a rotary evaporator at 50°C and dried in an oven at 40°C. The extract yields were calculated as a percentage of the dry extract weight relative to that of the initial plant material weight.

### 2.3 Antibacterial activity assay

Antibacterial activity was tested using the Kirby-Bauer disk diffusion method on Mueller-Hinton Agar (MHA) [16]. The extracts were prepared in dimethyl sulfoxide (DMSO) at concentrations of 250, 500, 750, and 1,000 mg/mL. Ciprofloxacin and 5% dimethyl sulfoxide (DMSO) served as positive and negative controls, respectively. Sterile paper discs (6 mm) were impregnated with 10 µL of each extract and placed on MHA plates inoculated with a bacterial suspension (McFarland standard, 0.5) [17]. The plates were then incubated at 37°C for 18–24 h. Each treatment was performed in triplicate. The inhibition zone accurately reflects the potential of the extract as an antibacterial agent [18]. The diameter of the inhibition zones was measured, and the level of inhibition was evaluated based on the following categories: no inhibition, 0 mm; weak inhibition, ≤5 mm; moderate inhibition, 6–10 mm; strong inhibition, 11–20 mm; and very strong inhibition, ≥21 mm [19].

A combination assay was conducted by mixing the most active extract with ciprofloxacin in a 1:1 volume ratio, following the same disc diffusion protocol.

### 2.4 GC-MS analysis

The most active extract was analyzed by gas chromatography-mass spectrometry (GC-MS) using a Thermo Scientific ISQ QD instrument equipped with an HP-5MS UI column (30 m long, 0.25 mm diameter, and 0.25 µm film thickness). Helium was used as the carrier gas at a flow rate of 1 ml/min. The temperature program began at 50°C (held for 2 min) and was increased to 280°C at a rate of 5°C/min. Mass spectra were obtained over the range of 40–500 amu. The identified compounds were matched with the NIST library based on their similarity index (SI). Mass spectral matching quality was interpreted based on similarity index (SI) or reverse similarity index (RSI) thresholds, where scores ≥900 indicated excellent matches, 800–900 indicated good matches, 700–800 indicated fair matches, and scores <600 indicated poor matches [20]. Only compounds with SI values >700 were considered for further analysis. 

### 2.5 PASS prediction and drug-likeness screening

The identified compounds were classified using the NP-Classifier (https://npclassifier.ucsd.edu/) [21] based on the SMILES data retrieved from PubChem (https://pubchem.ncbi.nlm.nih.gov/) [22]. The compounds were screened for biological activity using Way2Drug PASS Online (https://www.way2drug.com/passonline/) [23], and drug-likeness was evaluated based on Lipinski’s Rule of Five using SwissADME (https://www.swissadme.ch/) [24]. Compounds that met Lipinski’s criteria were selected for molecular docking analysis.

### 2.6 Preparation of proteins and ligands

Protein targets were selected based on their relevance to bacterial resistance: PBP2 (PDB ID: 6G9S) [10] and AcrB (PDB ID: 5NC5) [11]. The 3D structures of the proteins were obtained from the RCSB PDB database (https://rcbs.org) [25]. Protein structures were prepared using UCSF Chimera 1.17.3, and non-standard molecules such as ligands and water were removed. Native ligands were also collected from the RCSB PDB in SDF format. 

The 3D structures of the small molecules were obtained from PubChem (https://pubchem.ncbi.nlm.nih.gov) in SDF format. Ligands were prepared using OpenBabel integrated with PyRx, converted from SDF to PDBQT format by minimizing the energy with a universal force field (UFF), and converted to AutoDock ligands. The protein targets were inserted into AutoDock Vina Wizard, which was integrated with PyRx. This step automatically added hydrogen molecules, merged nonpolar hydrogen, and added Gasteiger charges to the structures, converting them into PDBQT format for docking [26].

### 2.7 Molecular docking: AutoDock Vina (PyRx)

Molecular docking was performed using AutoDock Vina Wizard integrated with PyRx. The docking grid box coordinates were determined based on the known binding sites of native ligands PUY and ET5, with the grid box centered on chain B of AcrB (X=26.514; Y=–52.578; Z=–51.185; dimensions: X=20.502; Y=12.060; Z=7.236) for PUY and on the PBP2 binding site (X=8.000; Y=40.000; Z=43.000; dimensions: X=16.800; Y=25.200; Z=12.000) for ET5. The binding affinity values (kcal/mol) were then determined. The best poses were visualized using PyMOL and Discovery Studio Visualizer 2024 Client to examine the interactions between the ligands and key amino acid residues.

### 2.8 Statistical analysis

The zone of inhibition data were analyzed using one-way ANOVA followed by Tukey’s post-hoc test for pairwise comparison (p<0.05, considered significant) using IBM SPSS Statistics 25.

## 3 Result and discussion

### 3.1 Yield and extraction efficiency

The clove buds extracted using ethanol yielded 27.44%, which was significantly higher than the 9.96% obtained from the n-hexane extraction (Table 1 (Tab. 1)). This suggests that the polar properties of ethanol are more effective in solubilizing secondary metabolites, such as flavonoids, alkaloids, tannins, and phenolic compounds, consistent with previous studies [27]. A higher yield also implies a greater concentration of potentially bioactive small molecules, which may contribute to antibacterial activity. 

### 3.2 Antibacterial activity of clove buds extracts

**Figure 1:**
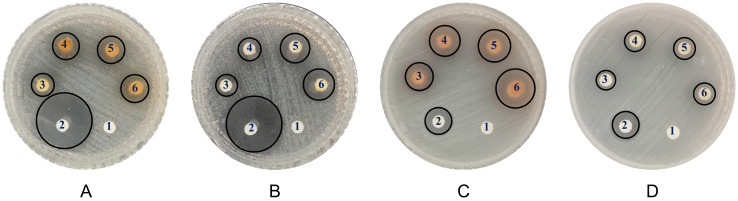
Results of antibacterial assays against *E. coli* ATCC (A, B) and MDR-UPEC (C, D) using ethanol extract (A, C) and n-hexane extract (B, D). (1) Negative control (5% DMSO); (2) positive control (ciprofloxacin 1 mg/mL); (3) 250 mg/mL; (4) 500 mg/mL; (5) 750 mg/mL; and (6) 1,000 mg/mL

The disc diffusion assay showed that both extracts inhibited *E. coli* ATCC and MDR-UPEC strains, with the ethanolic extract consistently demonstrating larger inhibition zones (Figure 1 (Fig. 1)). The antibacterial test results (Table 2 (Tab. 2)) demonstrated the efficacy of clove bud extracts against *Escherichia coli* ATCC and multidrug-resistant uropathogenic *E. coli* (MDR-UPEC). Ciprofloxacin, as a positive control, showed the largest inhibition zone against the ATCC strain (27.77±0.81 mm), categorized as very strong inhibition, whereas it produced a smaller zone against MDR-UPEC (9.47±0.49 mm, moderate category), indicating resistance of the MDR-UPEC isolate to ciprofloxacin. 

The ethanol extract of clove buds exhibited promising inhibitory effects, particularly against MDR-UPEC. At 250 mg/mL, it formed a 11.07±0.32 mm inhibition zone (strong category), which increased to 16.77±1.36 mm at 1,000 mg/mL concentration. ANOVA revealed a significant correlation between extract concentration and inhibition diameter (p=0.000). However, no significant differences were observed between the 500, 750, and 1,000 mg/mL concentrations. Interestingly, the ethanol extract outperformed ciprofloxacin against MDR-UPEC, suggesting that ethanol-extracted small molecules could serve as alternative antibacterial agents.

In contrast, the n-hexane extract exhibited weaker antibacterial activity, forming only a 4.43±0.85 mm zone against MDR-UPEC at its highest concentration, with no significant effect (p=0.235). This suggests that the nonpolar compounds in the extract are less effective against MDR-UPEC.

Against *E. coli* ATCC, the ethanol extract exhibited weak to moderate activity, with inhibition zones increasing from 3.83±2.08 mm to 8.33±0.76 mm. ANOVA indicated a significant difference (p=0.007), with the most notable increase observed from 250 mg/mL to 750 mg/mL of the extract. The n-hexane extract performed slightly better on ATCC than on MDR-UPEC, with a maximum inhibition zone of 6.23±1.19 mm, showing significant results (p=0.020). 

Overall, the ethanol extract consistently demonstrated better antibacterial activity than n-hexane, likely because of its ability to extract polar and semipolar bioactive compounds such as flavonoids, tannins, and phenolics [28]. Previous studies have established that phenolic compounds possess strong antibacterial properties against various bacterial pathogens [29]. Gonelimali et al. [30] also reported similar inhibition zones (13.2–17.4 mm) of clove ethanol extracts against *E. coli*. These findings highlight the potential of clove ethanol extract as a promising anti-MDR agent, justifying further analysis using GC-MS to identify the small molecules responsible for its activity in the future. 

**Figure 2:**
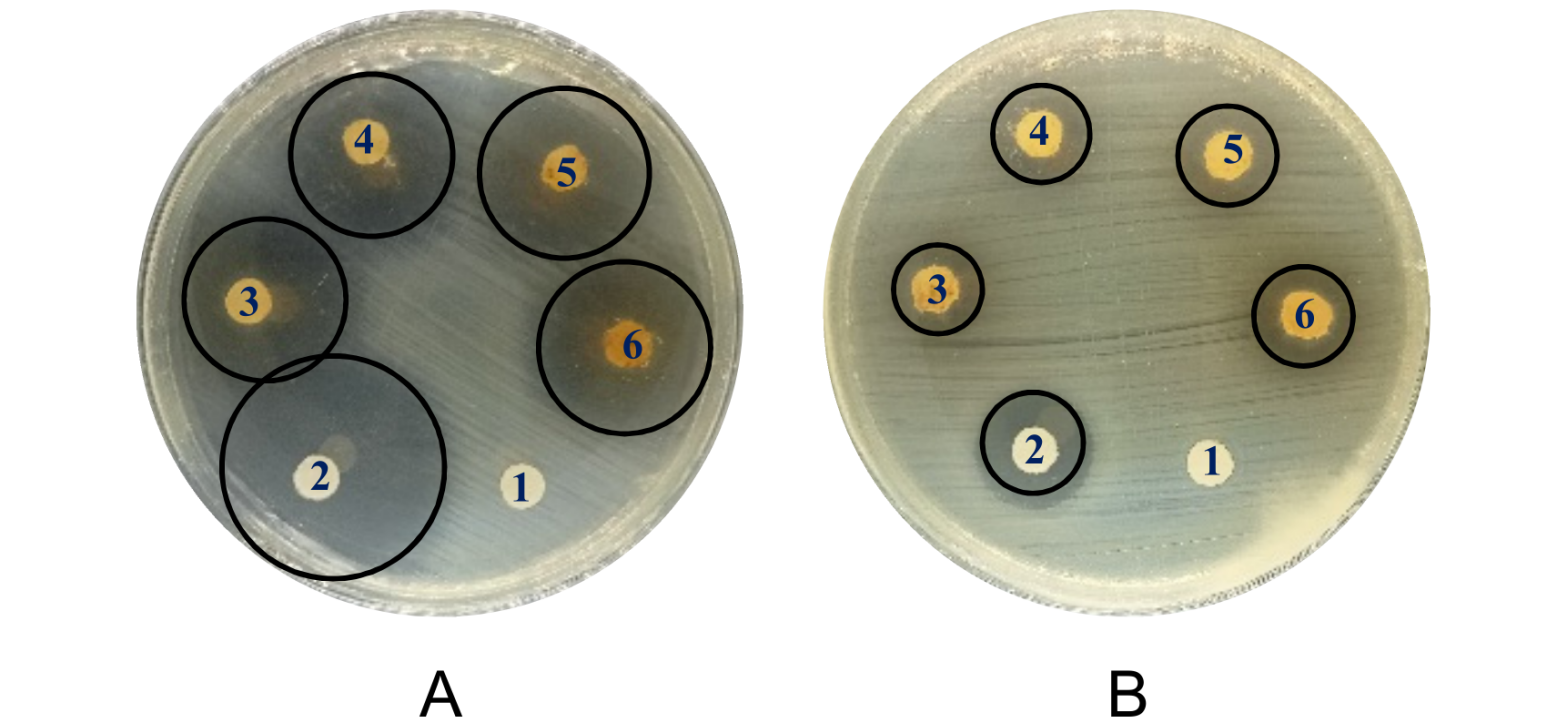
Antibacterial assay results of ethanolic extract combined with ciprofloxacin against *E. coli* ATCC (A) and MDR-UPEC (B). (1) Negative control (5% DMSO); (2) positive control (ciprofloxacin 1 mg/mL); (3) 250 mg/mL; (4) 500 mg/mL; (5) 750 mg/mL; and (6) 1,000 mg/mL

As shown in Figure 2 (Fig. 2) and Table 3 (Tab. 3), the combination of clove ethanolic extract with ciprofloxacin exhibited stronger antibacterial activity against* E. coli* ATCC than against MDR-UPEC. Although the combination resulted in moderate inhibition against MDR-UPEC (6.23±0.40 mm to 8.10±0.20 mm), the effect was significantly lower than that of ciprofloxacin alone (9.47±0.49 mm) or a single application of ethanol extract (11.07±0.32 mm), indicating an antagonistic interaction. In contrast, for *E. coli* ATCC, the combination produced inhibition zones of 17.80±0.26 mm to 18.83±0.32 mm, significantly higher than the extract alone but still lower than ciprofloxacin (27.77±0.81 mm), suggesting no synergistic interaction. 

According to [31], a synergistic effect occurs when the combined treatment exceeds the sum of the individual effects, whereas an antagonistic interaction occurs when the extract reduces the efficacy of the antibiotic. The observed antagonistic outcome may have resulted from interference of clove extract compounds with ciprofloxacin diffusion or activity, possibly limiting its effectiveness. This contrasts with the findings of [1], who reported an indifferent additive interaction using checkerboard assays. This difference may be attributed to the limitations of disk diffusion methods in representing true interactions, underscoring the need for complementary in silico studies to further explore the role of clove-derived small molecules in combating MDR-UPEC. 

### 3.3 GC-MS analysis and identification of small molecules

A total of 29 compounds were identified in the GC-MS analysis of the clove ethanolic extract (Table 4 (Tab. 4)), with the majority belonging to five major groups: phenylpropanoids, sesquiterpenoids, diterpenoids, fatty acids, and steroids. Eugenol (35.40%) was the most abundant compound, followed by cis-13-octadecenoic acid (19.04%), 3-allyl-6-methoxyphenyl acetate (14.12%), caryophyllene (5.65%), and octadecanoic acid (2.75%). Eugenol is known for its broad-spectrum antibacterial activity, including efficacy against antibiotic-resistant strains [15]. In silico predictions were performed using Way2Drug PASS Online and SwissADME to evaluate the pharmacological potential of these small molecules. 

### 3.4 PASS prediction and drug-likeness screening

PASS Online analysis, based on structure–activity relationships (SAR), was used to predict the antibacterial potential of the identified small molecules [32]. A probability of being active (Pa) value above 0.7 indicates a high likelihood of biological activity, while values between 0.5 and 0.7 suggest moderate potential, and values below 0.5 are considered low [33]. As shown in Table 5 (Tab. 5), SM10 emerged as the strongest antibacterial candidate, with a Pa of 0.659 and Pi (probability of being inactive) of 0.006, followed by SM5 (Pa=0.522; Pi=0.014), indicating its promising bioactivity. All other small molecules exhibited low predicted activities (Pa<0.5), necessitating further experimental validation. Notably, SM29 lacked predicted values, likely because of the limitations of structural recognition by the PASS algorithm. Interestingly, eugenol (SM8), a major component of clove extract, exhibited a relatively low Pa value of 0.325, contradicting previous reports [15], which confirmed its antibacterial mechanism of disrupting bacterial cytoplasmic membrane stability. This highlights the importance of validating computational predictions using experimental assays, particularly for dominant compounds.

Lipinski’s Rule of Five was applied to evaluate the oral drug-likeness of the identified small molecules based on key physicochemical parameters such as molecular weight (MW), lipophilicity (LogP), hydrogen bond donors (nHBD), and acceptors (nHBA) [34], [35]. The 14 compounds are listed in Table 6 (Tab. 6). (SM1–SM9, SM11, SM16, SM24, SM28, and SM29) fully complied with Lipinski’s criteria and were predominantly classified as sesquiterpenoids, phenylpropanoids, and steroids, indicating their strong potential as orally active drug candidates. These molecules were selected for molecular docking studies. 

### 3.5 Molecular docking with resistance-associated proteins

A molecular docking study targeted two proteins associated with multidrug-resistant uropathogenic *Escherichia coli* (MDR-UPEC). One of the main targets is penicillin-binding protein 2 (PBP2; PDB ID: 6G9S), which plays a crucial role in peptidoglycan crosslinking during cell wall synthesis [10], [36]. Inhibition of PBP2 disrupts cell wall integrity, leading to morphological changes in *E. coli* and transforming it into fragile spherical cells that are prone to lysis and unable to divide [37]. This weakened state potentially enhances the permeability of the bacterial membrane, thereby facilitating the entry of other small molecules that can further intoxicate and kill the cells. 

Another target of this study was AcrB (PDB ID: 5NC5), a critical component of the AcrAB-TolC efflux system that actively pumps antibiotics and toxic compounds out of bacterial cells [11]. PBP2 and AcrB were selected based on their central roles in the antibiotic resistance mechanisms of MDR-UPEC. Inhibiting the AcrB efflux pump is expected to prevent the extrusion of active compounds from bacterial cells, thereby increasing the intracellular accumulation of antibacterial agents and enhancing their effectiveness [11]. 

**Figure 3:**
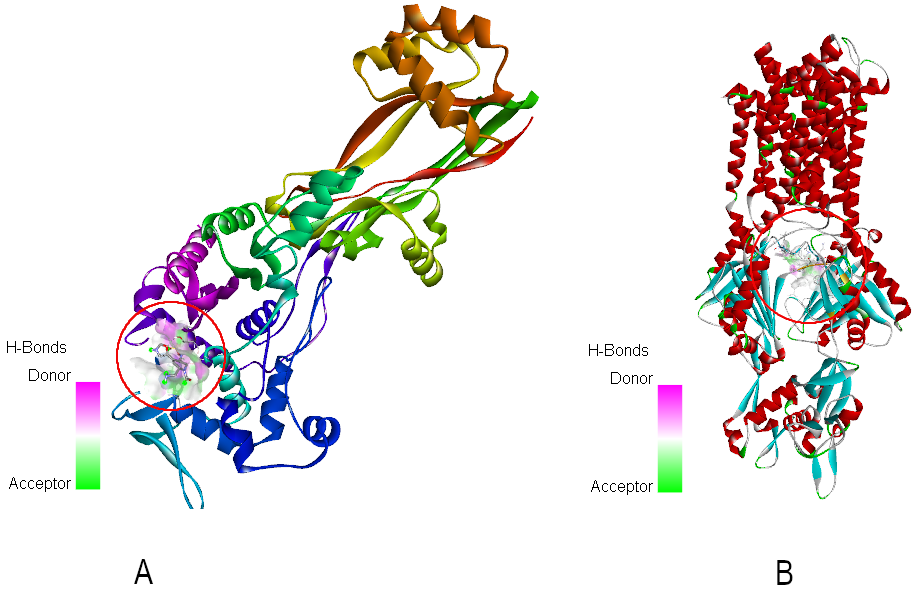
Binding site of ET5 in PBP2 (A) and PUY in AcrB (B)

A total of 14 small molecules identified from the ethanolic extract of clove and screened for druglikeness based on Lipinski’s rule were docked against both target proteins. ET5 and puromycin (PUY) were used as the native ligands for PBP2 and AcrB, respectively. The binding positions of the native ligands within the active sites are shown in Figure 3 (Fig. 3). 

**Figure 4:**
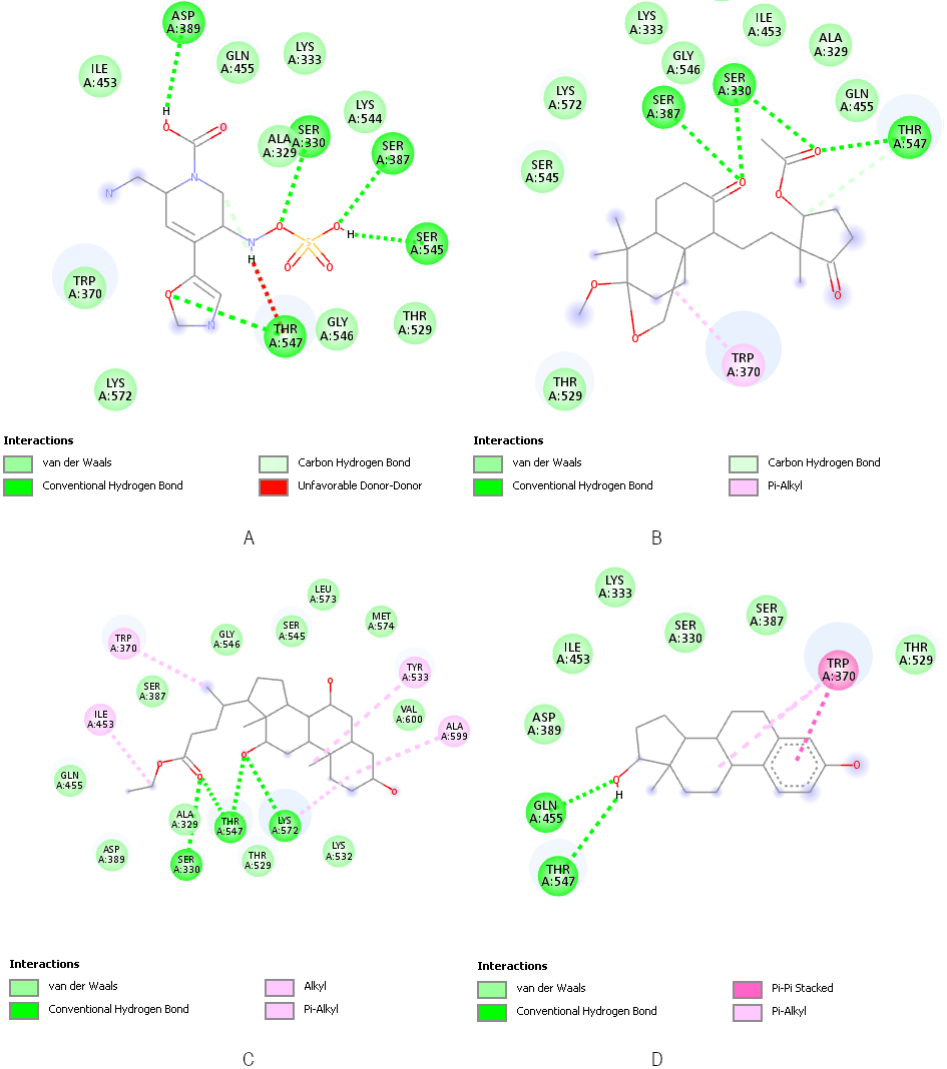
Docking visualisation of ET5 (A), SM7 (B), SM28 (C) and SM29 (D) to PBP2

Docking simulations using AutoDock Vina within PyRx (Table 7 (Tab. 7)) revealed that three small molecules, SM7, SM28, and SM29, exhibited higher binding affinities to PBP2, ranging from –8.0 to –7.6 kcal/mol, compared to the native ligand ET5 (–6.9 kcal/mol). Negative binding affinity values indicate favorable interactions between the ligand and target macromolecule, with more negative values suggesting stronger and more stable predicted binding [38]. These results suggest that SM7, SM28, and SM29 have a higher potential to interact with and inhibit PBP2 than the reference ligand. These ligands share key hydrogen bonding interactions with PBP2 residues involved in ET5 binding, such as THR547 and SER330 (Figure 4 (Fig. 4)), indicating similar binding modes. Although they establish fewer hydrogen bonds than ET5, their stable binding is largely supported by van der Waals forces and hydrophobic interactions. 

**Figure 5:**
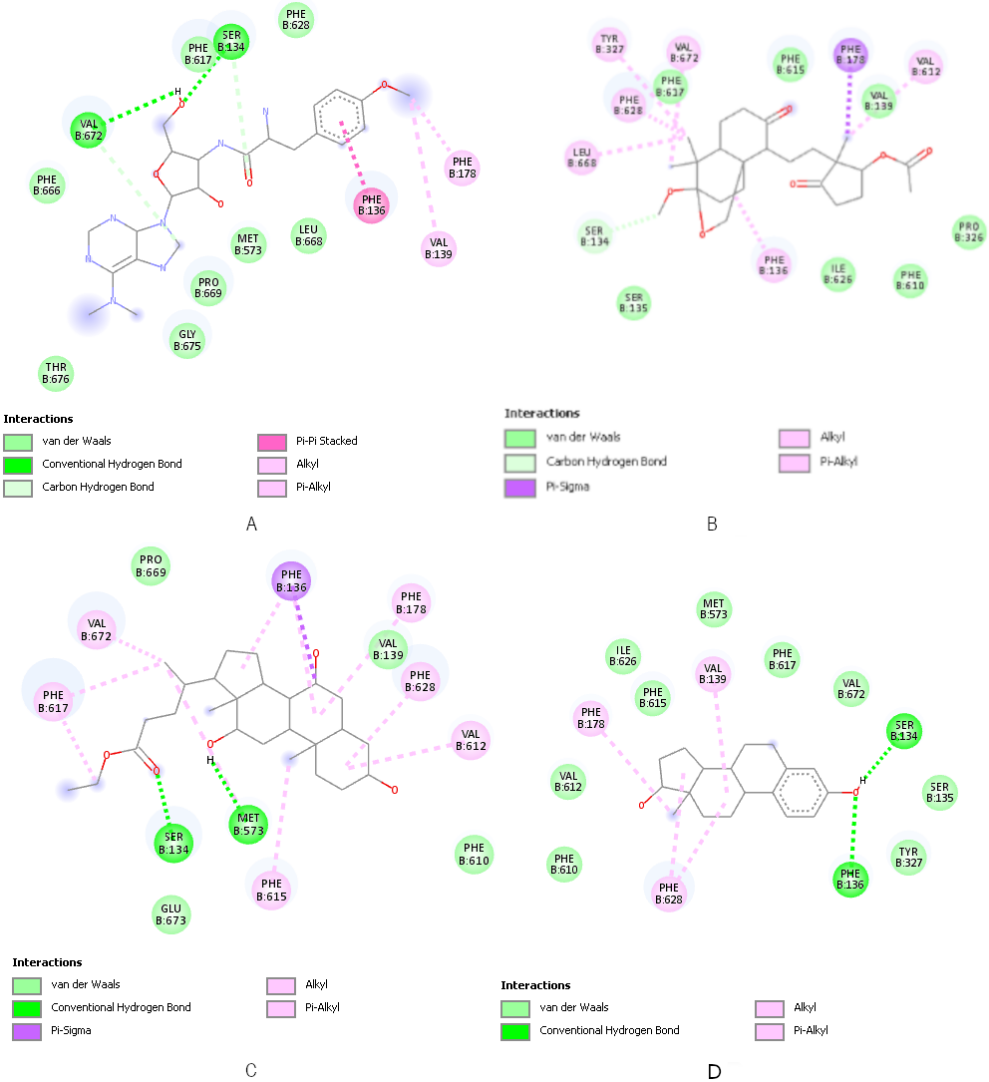
Docking visualisation of PUY (A), SM7 (B), SM28 (C) and SM29 (D) to AcrB

In the AcrB docking simulations (Table 7 (Tab. 7)), the ten small molecules exhibited stronger binding to the target protein than the native ligand PUY (–6.5 kcal/mol). The top three small molecules were SM7 (–9.3 kcal/mol), SM28 (–8.5 kcal/mol), and SM29 (–8.6 kcal/mol). SM28 and SM29 also replicated the key hydrogen bond interactions with SER134 and PHE136, which is consistent with PUY’s binding profile of PUY (Figure 5 (Fig. 5)). These results suggest a dual inhibitory potential against both cell wall synthesis (PBP2) and efflux mechanisms (AcrB) in MDR UPEC.

Despite their strong docking performance, PASS Online predictions showed low Pa values (<0.5) for these compounds, highlighting the importance of experimental validation. Ethyl isoallocholate (SM28) has been reported to inhibit *Pseudomonas aeruginosa* [39]. The strong binding affinity of this compound to the five pathogenic receptors observed in this study further supports its potential as an effective antibacterial agent. The antibacterial potential of 8,14-Seco-3,19-epoxyandrostane-8,14-dione, 17-acetoxy-3β-methoxy-4,4-dimethyl-, and Estra-1,3,5(10)-trien-17β-ol remains poorly explored, indicating the need for further identification and characterization.

SM7, SM28, and SM29, identified as promising small molecules, were detected in trace amounts (0.06–0.11%) in the GC-MS profile, suggesting that the overall antibacterial effect of the extract may also involve synergistic interactions with dominant compounds such as eugenol (35.40%) and caryophyllene (5.65%). Although these major constituents exhibit weaker binding, they may enhance cellular permeability or contribute to indirect effects.

## 4 Conclusion

This investigation revealed that the ethanol extract exhibited inhibitory activity against MDR-UPEC, producing inhibition zones ranging from 11.07±0.32 to 16.77±1.36 across tested concentrations in a disk diffusion assay. However, their combination with ciprofloxacin resulted in an antagonistic interaction. GC-MS analysis of the ethanol bud extract identified 29 small molecules, of which three showed potential inhibition of PBP2 and ten showed affinity toward AcrB. Notably, 8,14-Seco-3,19-epoxyandrostane-8,14-dione, 17-acetoxy-3β-methoxy-4,4-dimethyl- (SM7), ethyl iso-allocholate (SM28), and Estra-1,3,5(10)-trien-17β-ol (SM29) exhibited strong binding affinities for both PBP2 and AcrB target proteins. Their dual-targeting ability makes them promising candidates for further research. Future research should focus on compound isolation, in vitro and in vivo validation, and structure-based optimization for the potential development of standardized antibacterial agents.

## Notes

### Funding

We thank Universitas Gadjah Mada for supporting this research under RTA grant 4193/UN1.P1/Dit-Lit/PT.01.03/2025.

### Competing interests

The authors declare that they have no competing interests.

## Figures and Tables

**Table 1:**
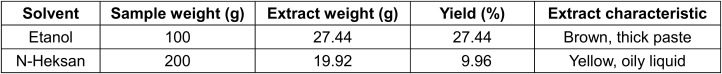
Yield of clove extracts using two different solvents

**Table 2:**
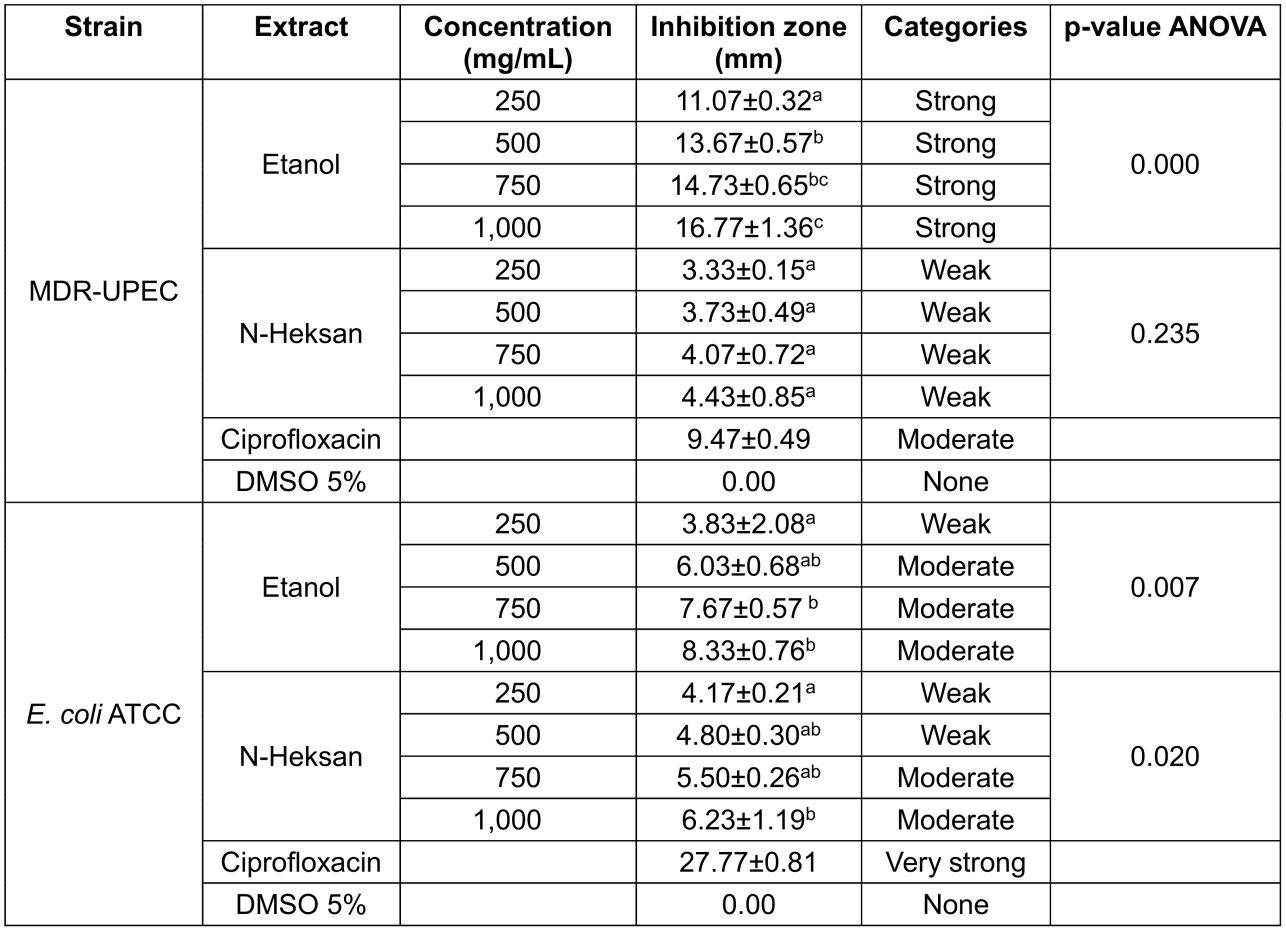
Antibacterial activity test

**Table 3:**
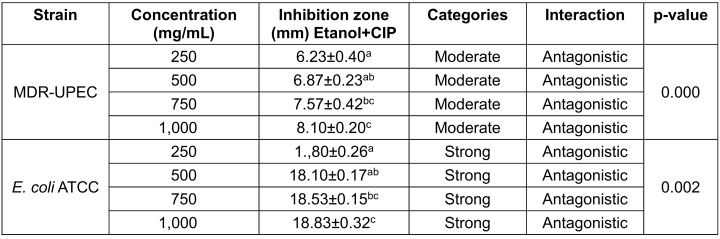
Antibacterial activity test of ethanolic extract combined with ciprofloxacin

**Table 4:**
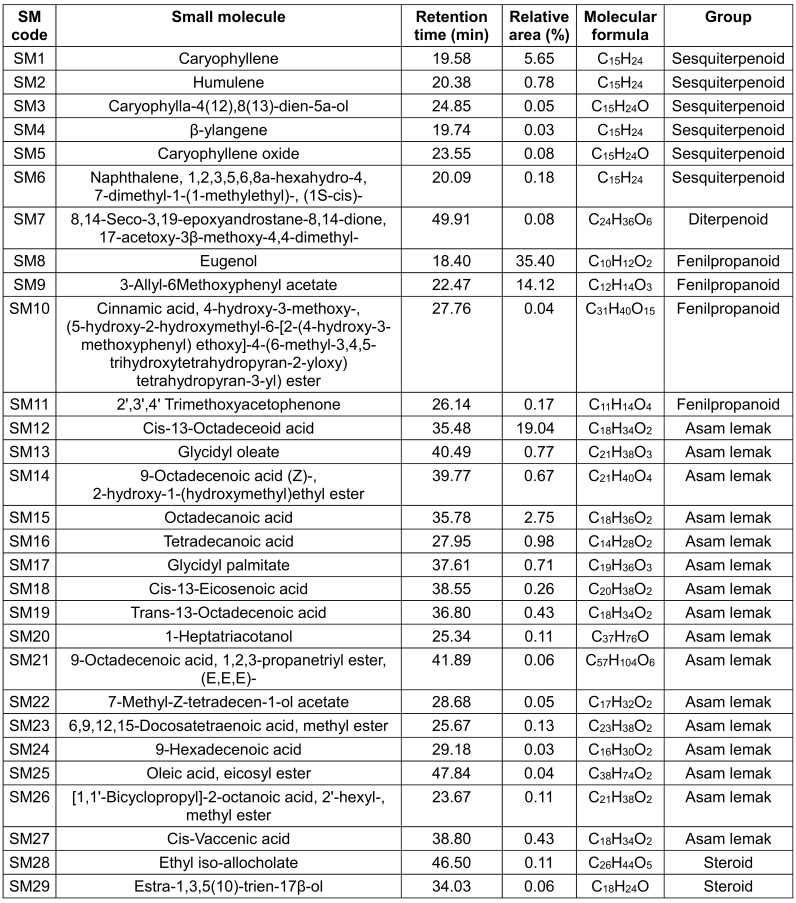
GC-MS analysis of small molecules in ethanolic extract

**Table 5:**
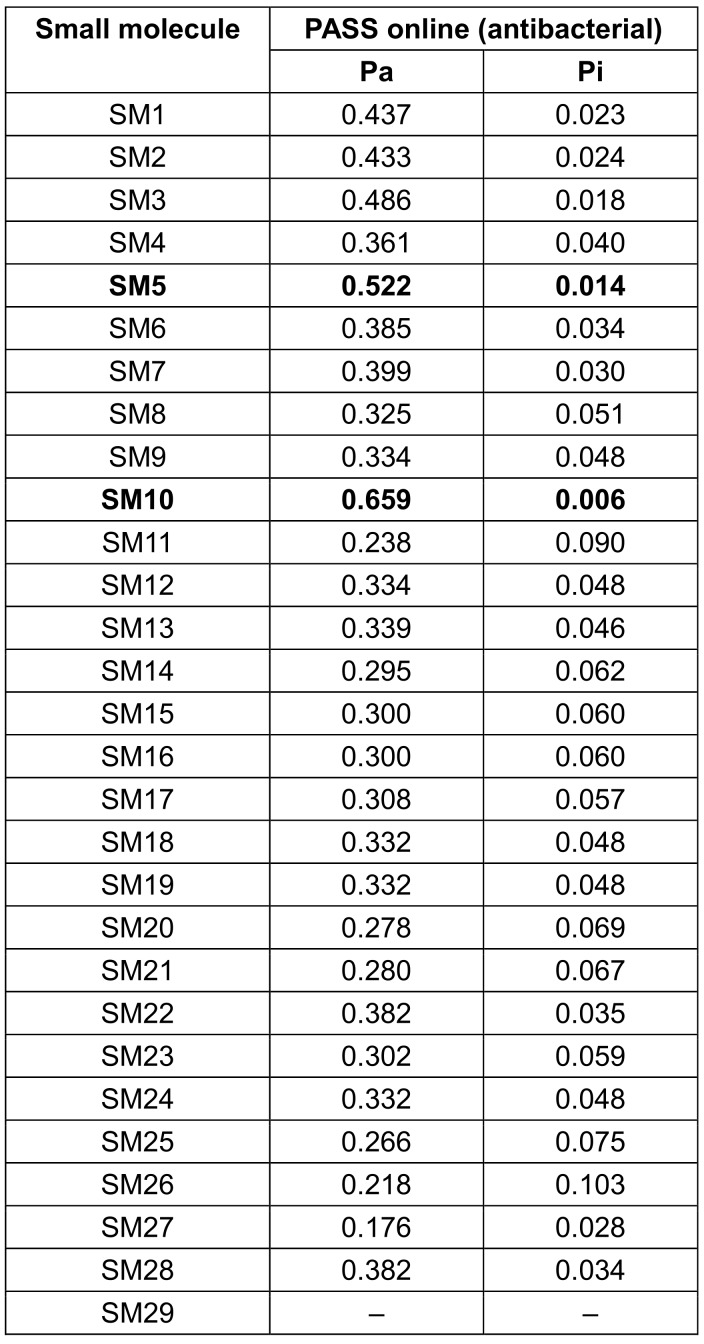
PASS online prediction of antibacterial activities of small molecules from clove ethanolic extract

**Table 6:**
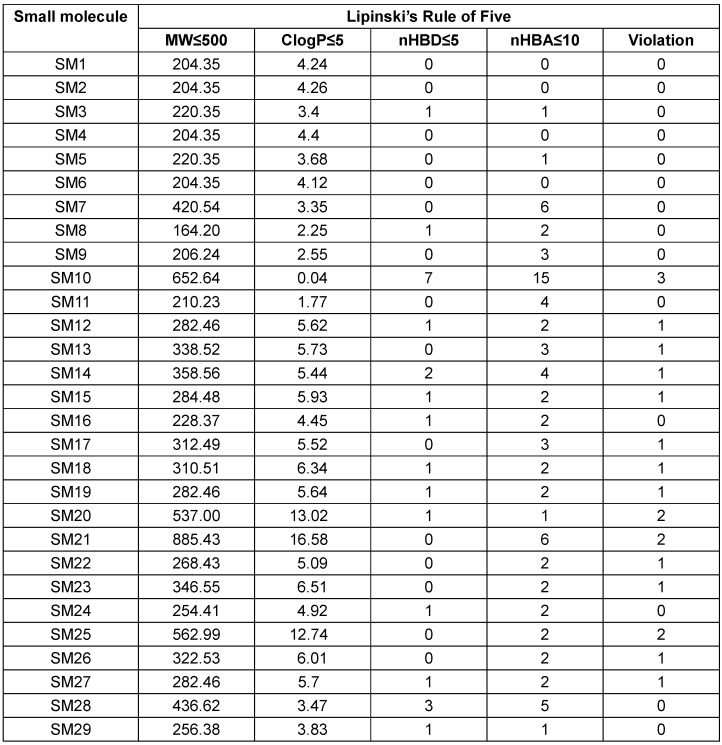
Drug-likeness evaluation of small molecules based on Lipinski’s Rule of Five

**Table 7:**
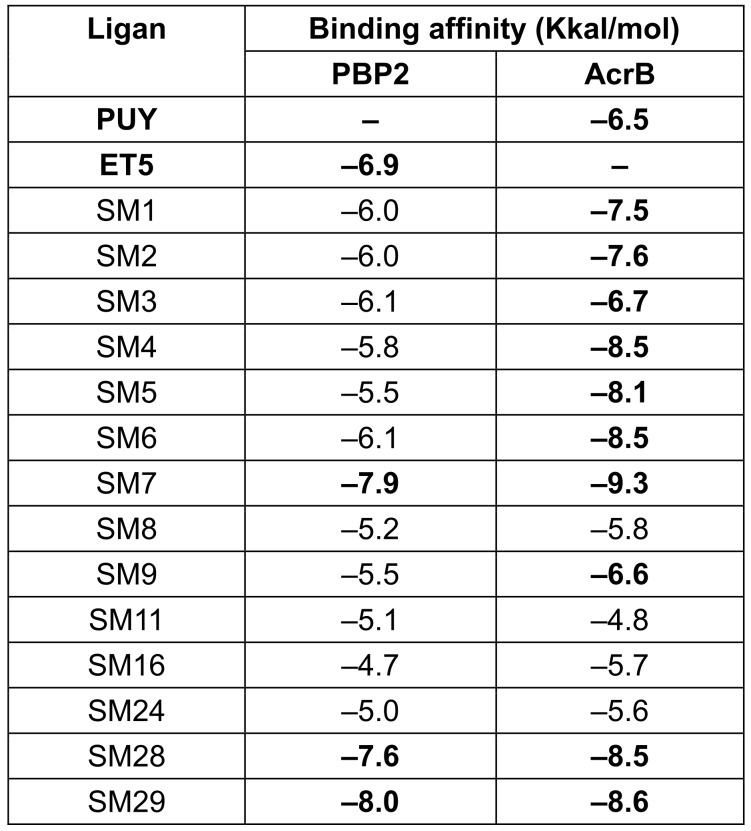
Binding affinity of small molecules from ethanolic clove extract docked to AcrB and PBP2
